# The Recommendations for the Management of Chinese Children With Epilepsy During the COVID-19 Outbreak

**DOI:** 10.3389/fped.2020.00495

**Published:** 2020-08-25

**Authors:** Baiyu Chen, Miriam Kessi, Shimeng Chen, Juan Xiong, Liwen Wu, Xiaolu Deng, Lifen Yang, Fang He, Fei Yin, Jing Peng

**Affiliations:** ^1^Department of Pediatrics, Xiangya Hospital, Central South University, Changsha, China; ^2^Hunan Intellectual and Developmental Disabilities Research Center, Changsha, China

**Keywords:** COVID-19, SARS-CoV2, epilepsy, children, management recommendations

## Abstract

The coronavirus disease (COVID-19) is the most severe public health problem facing the world currently. Social distancing and avoidance of unnecessary movements are preventive strategies that are being advocated to prevent the spread of the causative virus [severe acute respiratory syndrome (SARS)-CoV2]. It is known that epileptic children need long term treatments (antiepileptic drugs and/or immunosuppressive agents) as well as close follow up due to the nature of the disease. In addition, it is clear that epilepsy can concur with other chronic illnesses which can lower body immunity. As a result, epileptic children have high risk of acquiring this novel disease due to weak/immature immune system. Of concern, the management of children with epilepsy has become more challenging during this outbreak due to the prevention measures that are being taken. Although children with controlled seizures can be managed at home, it is challenging for pediatricians when it comes to cases with uncontrolled seizures/severe cases. To this end, we provide recommendations for the management of epileptic children at home, outpatient and inpatient settings.

## Introduction

The novel coronavirus outbreak was observed for the first time at Wuhan, Hubei province in China on December 2019, however, it has now become a pandemic (1). The virus was named as a severe acute respiratory syndrome coronavirus 2 (SARS-CoV-2) by the international committee on classification of viruses. Meanwhile, the WHO named the resulting illness as a corona virus disease-19 (COVID-19), which was listed as a class B infectious disease (2, 3). This infection can be transmitted through respiratory droplets, aerosol particles and contact (4–6). As of April 4, 2020, a total number of 1,085,730 cases were confirmed to be infected globally, 59,072 of whom died (7). All age groups are susceptible (8).

COVID-19 is characterized by mild upper respiratory tract symptoms including fever, headache, sore throat, cough and nasal congestion, however, some cases present with severe illnesses that progress from pneumonia, respiratory failure and death (8, 9). 1–5% of diagnosed COVID-19 cases are children according to the systematic review (10). They may be asymptomatic or present with fever, dry cough, nasal congestion, runny nose, fatigue, abdominal discomfort, nausea, vomiting, and diarrhea (11). Fortunately, they seem to have mild disease course and better prognosis than adults despite of their immature/weak immune systems (10).

Childhood epilepsy is a common chronic neurological disease that need long term treatment (antiepileptic drugs and/immunosuppressive agents) and close follow up (12). One study suggested that epileptic patients are neither more likely to be infected by the virus nor have severe COVID-19 manifestations because of epilepsy (13). Nevertheless, epilepsy can co-exist with other chronic diseases that can lower body immunity such as immune disorders, malnutrition/eating difficulties, nutritional deficiency, immobility, respiratory conditions (including asthma), diabetes mellitus, hypertension, and severe heart disease (14–17). Thus, epileptic children might be more susceptible to the current outbreak due to immunosuppressive agents and/or co-existing health conditions. How to maintain safety and well-being of epileptic children while preventing the transmission of SARS-CoV2 is challenging to pediatricians. Therefore, we propose a strategic plan for the management of children with epilepsy during the outbreak.

## Methods

These recommendations were made by senior pediatric neurologists of Xiangya Hospital, Central South University according to their expert opinions and current literatures.

## Outpatient Management

It is important to reduce children's outings and gatherings as the process of preventing cross-infection. However, this strategy has negative consequences on children with chronic diseases who need not only adjustment of the drug (s)/dosage (s) but also long-term medication (s)/follow up according to the course of the disease. This strategy challenges the work of pediatricians too as they are responsible for ensuring safety and well-being of epileptic children. On the one hand, children and their parents/guardians might delay to go to the hospital for follow up, consequently, they might be unable to continue with the scheduled treatments or fail to receive adjustment of drugs/dosages timely. On the other hand, children and parents/guardians might increase self-prescription behaviors including reducing the amount/dosages, changing the type of medicines when there is insufficient antiepileptic drugs, using family-owned/adult medicines for epilepsy or other illnesses, and some might stop the medications completely. All aforementioned issues can increase the malpractice emergency medical risks. How to conduct epilepsy outpatient clinic and emergency services concurrently is a problem that draws pediatricians' attention. Here we list strategies that can be utilized.

### Construction of COVID-19 Related Medication Database for Children With Epilepsy

We recommend pediatric neurologists to construct a COVID-19 related medication database for monitoring the response and adverse reactions. Those medications should be administered by the doctors or trained nurses at the hospital. Recommended drugs for trials include atazanavir (ATV), darunavir/cobicistat (DRV/c), lopinavir/ritonavir (LPV/r), remdesivir/GS-5734(RDV), favipiravir (FAVI), chloroquine (CLQ), hydroxychloroquine (HCLQ), nitazoxanide (NITA), ribavirin(RBV), tocilizumab (TCZ), interferon β-1a (IFN-β-1a), oseltamivir (OSV) (https://www.lice.it/pdf/Antiepileptic_drugs_interactions_in_COVID-19.pdf) (18). Since some antiepileptic drugs (AEDs) have strong interactions with anti-COVID-19 drugs, and can induce or inhibit cytochromes, attention should be paid on issues such as compatibility, contraindications, usage and dosage.

#### AEDs That Should Not Be Co-administered With Anti-COVID-19 Drugs

For the epileptic patients who are affected with COVID-19, medications such as ATV, DRV/c, RDV, CLQ, and HCLQ should not be co-administered with carbamazepine, phenytoin, phenobarbital, and primidone. The use of carbamazepine, phenytoin, phenobarbital and primidone will potentially decrease exposure of anti-COVID drugs. Moreover, the use of ATV and DRV/c can potentially increase exposure of the carbamazepine. Besides, the use of DRV/c can potentially decrease exposure of phenobarbital (18).

#### Potential Drug Interaction Which May Require a Dose Adjustment or Close Monitoring

Some AEDs and anti-COVID-19 medications have potential interactions which may require a dose adjustment or close monitoring. Cenobamate, eslicarbazepine, oxcarbazepine, and rufinamide can decrease exposure of ATV.

Cenobamate, eslicarbazepine, felbamate, oxcarbazepine, perampanel, rufinamide, topiramate, and valproic acid can decrease exposure of DRV/c. AEDs that can decrease exposure of LPV/r include: carbamazepine, cenobamate, eslicarbazepine, oxcarbazepine, phenytoin, phenobarbital, primidone, and rufinamide. AEDs that can reduce exposure of RDV include: eslicarbazepine, oxcarbazepine, and rufinamide. AEDs that can decrease exposure of CLQ and HCLQ include: cenobamate, eslicarbazepine, oxcarbazepine, and rufinamide.

The use of ATV, DRV/c, and LPV/r can potentially increase exposure of the clonazepam, clobazam, diazepam, sulthiame, and tiagabine. The use of DRV/c, LPV/r, CLQ, and HCLQ can potentially increase exposure of the cannabidiol. The use of ATV and LPV/r can potentially increase exposure of the perampanel. The use of DRV/c can potentially increase exposure of the lamotrigine. The use of ATV, LPV/r, CLQ and HCLQ can potentially decrease exposure of the felbamate. The use of DRV/c can potentially decrease exposure of the oxcarbazepine. The use of LPV/r can potentially decrease exposure of the primidone. The use of ATV has no significant effect on cannabidiol. The use of IFN-β-1a^4^ has no significant effect on carbamazepine, cannabidiol, felbamate, oxcarbazepine, phenytoin, phenobarbital, and valproic acid (18). Thus, we advise clinicians to adjust dosage according to the specific drug interaction.

#### Potential Interaction Likely to Be of Weak Intensity

Some AEDs and anti-COVID-19 medications have weak intensity interactions, as a result, additional acts/monitoring or dosage adjustment are not mandatory. The use of TCZ can potentially decrease exposure of the carbamazepine, phenytoin, phenobarbital, and primidone. The use of RBV can potentially increase exposure of the brivaracetam. The use of LPV/r can potentially decrease exposure of the brivaracetam. The use of ATV has no significant effect on brivaracetam and lacosamide. The use of LPV/r has no significant effect on lacosamide (18).

#### No Clinically Significant Interaction Expected

The use of ATV has no significant effect on gabapentin, lamotrigine, levetiracetam, lorazepam, pregabalin, retigabine, topiramate, valproic acid, vigabatrin, and zonisamide. The use of DRV/c has no significant effect on brivaracetam, gabapentin, lamotrigine, levetiracetam, pregabalin, retigabine, and vigabatrin. The use of LPV/r has no significant effect on gabapentin, levetiracetam, lorazepam, pregabalin, retigabine, topiramate, vigabatrin, and zonisamide. The use of RDV has no significant effect on brivaracetam, cannabidiol, cenobamate, clonazepam, clobazam, diazepam, ethosuximide, felbamate, gabapentin, lacosamide, lamotrigine, levetiracetam, lorazepam, perampanel, pregabalin, retigabine, sulthiame, tiagabine, topiramate, valproic acid, vigabatrin, and zonisamide. The use of CLQ and HCLQ has no significant effect on clonazepam, clobazam, diazepam, ethosuximide, gabapentin, lacosamide, lamotrigine, levetiracetam, lorazepam, perampanel, pregabalin, retigabine, sulthiame, tiagabine, topiramate, valproic acid, vigabatrin, and zonisamide. With exception of phenytoin, the use of NITA has no significant interaction with AEDs. Except for brivaracetam, the use of RBV has no significant interaction with AEDs. Apart from carbamazepine, phenytoin, phenobarbital, and primidone, the use of TCZ has no significant interaction with AEDs. The use of FAVI and OSV has no significant interaction with AEDs (18).

### The Combination of Online and Offline Outpatient Medical Services Should Be Employed

Online service/telemedicine is the type of service which allows real time interactive communication between the patient, and clinician at the distant site. Electronic communication via phones and internet/social media are used to facilitate this kind of service. This service is cost-effective alternative to the traditional face-to-face (offline) way; face-to-face consultations or examinations between doctor and patient. Creation of an outpatient model that comprise the combination of online and offline services has become easy with an advancement of technology. The telemedicine service model is not limited by factors such as time and region, and can facilitate the process of children and parents in obtaining medical information and physician's help in a timely manner. Thus, we recommend an execution of such model in order to improve the efficiency and quality of the services.

### Implementation of Outpatient Medical Service Grading Strategy

There are several medications for epilepsy which are very heterogeneous and the prescription varies for each individual due to high risk of drug interactions. Therefore, pediatricians should consider the child's condition more comprehensively, and need to implement the most suitable individualized service. The services can be provided according to the grades as outlined below. Noteworthy, the below listed grades are more local/applicable in China only. Besides, this distinction is proposed only in time of COVID-19 outbreak to prevent the spread of this condition. However, other countries can adopt the same distinction for the same purpose.

#### Primary Medical Services

Primary medical services should be provided for the patients with the following features: [1] have good drug compliance, [2] have simple medication plans, [3] can receive continuation drugs at local outpatient clinics, [4] have controlled seizures. We recommend them to receive drugs at local hospitals while continuing with observation at home as well as online medical consultations whenever necessary.

#### Secondary Medical Services

We recommend secondary medical services for patients characterized by: [1] relatively controlled seizures but the drugs being used are not available in local clinics or pharmacies, [2] utilization of relatively complex and specific drugs, [3] previous history of adverse drug reactions, [4] previous history of poor drug compliance. Children with aforementioned criteria can be observed at home while participating in online medical services. Consultants should provide an extra care by instructing them on how to buy drugs online and use them properly. Nevertheless, we advise them to go to the hospital for treatment as soon as they experience any unusual attacks.

#### Tertiary Medical Services

Tertiary medical services should be provided for the children that meet the following criteria: [1] history of recurrent poor drug compliance, [2] using drugs related to severe complications, [3] using drugs accompanied by liver/kidney dysfunctions, [4] experiencing changes of the condition, [5] the existing treatment plans are not effective. Pediatricians are advised to combine the medical history, drug history, and necessary auxiliary examination results to determine whether patients with non-effective drugs/treatment plans need outpatient or inpatient services. We recommended urgent visit to the local hospital for those cases.

### Outpatient Service Management

Patients with the need to attend outpatient clinic should make an appointment through the phone/internet. We advise one person to accompany each patient according to the appointment schedule. Outpatient medical staffs should provide masks for children and their accompanying person. Thereafter, epidemiological history, presenting symptoms and body temperature should be recorded. If the children and their accompanying person have normal body temperature and don't have epidemiological history suggesting the risk of the COVID-19, they should be attended at the epilepsy outpatient clinic. Conversely, if the child has epidemiological history and/or respiratory symptoms suggestive of this illness, they should be directed to the specific outpatient clinic for the suspected cases of COVID-19 for further evaluation. For detailed diagnostic criteria, please refer to the Children's 2019 COVID-19 Diagnosis and Treatment Guidelines (Second Edition) (19).

### Electroencephalogram (EEG) Monitoring Management

The COVID-19 healthcare crisis and the resulting “stay-at-home” orders have created many obstacles for pediatricians. This is particularly true for the epileptic patients with uncontrolled seizures who often require frequent ambulatory visits, ancillary tests such as EEG, imaging, laboratory results, and even inpatient monitoring. The American Epilepsy Society statement on COVID-19 advises epileptic centers to utilize remote care options such as telephone, telehealth, and electronic health record messaging to manage their patients, a treatment model that is largely new to epilepsy care. International League Against Epilepsy advises pediatrician to counsel patients that they could experience an increase in seizures frequency due to decreased access to AEDs and an increased stress during COVID-19 (20). Therefore, strategies on how to perform EEG examination is particularly important during the epidemic.

In New York City, hospitals canceled all elective admissions to the epilepsy monitoring unit (EMU). As a result, the use of long-term continuous EEG (cEEG) monitoring in neurological and medical intensive care units (ICUs) decreased dramatically (21). The floor and ICU beds were occupied by COVID-19 patients, and the residents and attending physicians were instructed to use a portable 25 min emergency EEG as a substitute for cEEG study if indicated. The rationale for doing so was to reduce the risk of exposure to coronavirus to the neurophysiology technician. Either reusable or disposable electrodes were placed according to the international 10–20 system, and the technician stayed in the patient's room throughout the duration of the 25-min study. Once the technician returned to the EEG lab, the machine was cleaned with a commercially available germicidal disposable wipe which is bactericidal, tuberculocidal, and virucidal in 2 min prior to next use (21). The RNS System is a direct brain-responsive neuromodulation system that is a novel approach for tracking, and understanding patients' electrographic seizures, thus it is particularly important in COVID-19 healthcare provision (20).

In most cases, diagnostic EEG can be delayed to a later date. However, urgent situations/conditions including status epilepticus, electrical status epilepticus of slow sleep, non-convulsive status epilepticus, or infantile spasms (although video diagnosis of spasms by an experienced pediatric neurologist might be enough to initiate treatment if the risk of hospital attendance outweighs benefit) may require EEG. In some cases (e.g., infantile spasms), a brief outpatient study can provide critical information with low risk if precautions are used (13). Noteworthy, hyperventilation should be avoided during the EEG exams (22).

A system that reduce direct exposure of health care professionals to epileptic patients is advisable. For example, a clinic or hospital service can have a “neurologist” of the day or a technician performing all EEGs during a certain period and in a single environment. This will facilitate tracking of contacts, and minimize disruption should individual health professionals become infected. In managing status epilepticus, additional precautions should be undertaken to prevent airborne spread from secretions (13).

## Inpatient Service Management

First of all, pediatricians should constantly update themselves on the latest information and the measures that are being taken for the diagnosis and treatment of this illness. They should further conduct regular evaluations in the wards to pick new cases that develop symptoms in the hospital. Second, the hospital should establish a COVID-19 professional committee to conduct multidisciplinary consultations via internet so as to make good medical decision. Third, conventional management strategies should be adopted in order to minimize cross-infection between all people within the pediatric neurology ward. Besides the conventional hospital ward management strategy, no one in the pediatric neurology ward should enter other medical treatment areas without permission. We advise each hospital to create four zones in order to screen patients who are potentially infected but at the same time trying to minimize cross infection (23). Zone 1 should be the COVID-19 confirmed quarantine zone which can be used to treat confirmed cases. Zone 2 should be the COVID-19 suspected quarantine zone which can be used to observe and treat highly suspected cases. Zone 3 should deal with febrile children with low level of suspicion. Zone 4 should be pediatric neurology ward which can be used for the treatment of epileptic patients without COVID-19. Medical staff should raise awareness of COVID-19 epidemic prevention and control measures, limit the number of escorts, and prohibit unnecessary visits. Medical staff should monitor the temperature of inpatients and their escorts daily, strengthen health education, and provide proper hygiene advises including hand washing methods, cough etiquette and how to wear and handle masks properly. Medical staff should put on protective gears such as isolation suits, helmets, goggles and others.

## Specific Recommendations for Epileptic Syndromes

### Recommendations for Dravet Syndrome

Treatment options that seem to be effective in Dravet syndrome include sodium valproate, clobazam, stiripentol, topiramate, cannabidiol, fenfluramine, and ketogenic diet (24). Fortunately, most of these drugs can be taken orally. Thus, it seems telemedicine can help this group of patients, therefore, we recommend it.

### Recommendations for Lennox-Gastaut Syndrome

The recommended first-line drug option is sodium valporate. Other AEDs which can be considered include lamotrigine, rufinamide, topiramate, clobazam, and cannabidiol (25). Since most of these drugs can be taken orally, we recommend the use of telemedicine.

### Recommendations for Infantile Spasms (ISs)

The first-line drugs for ISs include adrenocorticotropic hormone (ACTH), vigabatrin (VGB) and corticosteroid (26). Oral prednisolone is considered to be effective too (26). Therefore, we recommend the utilization of oral VGB or prednisolone according to the etiology instead of ACTH during the outbreak. Besides, detailed recommendations for this condition during COVID-19 outbreak has been summarized recently (27).

### Recommendations for Continuous Spike-Wave During Slow Wave Sleep and Landau-Kleffner Syndrome

Recommended first-line drugs include valproate, ethosuximide, and benzodiazepine. For cases who are refractory to aforementioned AEDs, ACTH, or intravenous immunoglobulin (IVIg) tend to be used. Additional treatment modalities include ketogenic diet and surgery (28). Consequently, we recommend the trial of oral therapies first via telemedicine. The administration of ACTH or IVIg can be done in the hospital for the refractory cases.

### Recommendations for Ohtahara Syndrome

Corticosteroids and levetiracetam are the recommended first-line drugs. Adjuvants treatment options include zonisamide, vigabatrin, phenobarbital, and ketogenic diet (29). Fortunately, most of the above listed drugs can be given orally. Therefore, online outpatient service might be helpful for less severe cases.

## Family Management

Family management is crucial during this epidemic as most epileptic children are required to go to the hospital for follow-up visits but some might fail to do. We have listed recommendations below that can aid in ensuring the safety and well-being of epileptic children.

### Use of Antiepileptic Drugs

Although it might be difficult for the patients to return to the clinic as scheduled, they are advised to take their anti-epileptic drugs on time and in the right amount. Patients should not reduce or stop the drug without the evaluation of the doctor. Parents/guardians are encouraged to purchase drugs at the nearest local hospital/pharmacy, or through the official hospital information network whenever necessary. Noteworthy, some older children can take medication by themselves but with poor compliance (they might hide or throw drugs away) while others can overdose themselves by taking a lot of medication (s) when they are pessimistic or impulsive. Therefore, parents/guardians should observe their children closely as they take drugs, ensure safe storage and check the remained amount regularly, and telehealth should be utilized whenever possible, using video ideally, or phone if video is not accessible. Such contact with patients and parents/guardians can alleviate their anxiety and concerns, and remind patients and their parents/guardians of the importance of current dosage and precise dosage.

### Observe Changes of the Patients' Condition Closely and Prevent Accidents

Older epileptic children might be more prone to stress reactions, such as nervousness, anxiety, and anger during this epidemic. Therefore, parents/guardians should closely observe changes in children's emotions, thoughts, and behaviors, and respond to them in a timely manner. If the child has signs of pessimism/impulse, parents should monitor them closely 24 h a day and manage the dangerous goods in the house to prevent accidents.

### Maintain a Healthy Regular Daily Life

Children are more likely to acquire bacterial and viral infections if they become too lazy to the extent of ignoring the personal hygiene. Parents/guardians should urge children to brush their teeth, wash their hands, cut their nails frequently, and bathe regularly. In addition, they should ensure that children receive a balanced diet, supplemented with sufficient water and fruit juices which cannot be replaced with beverages such as coffee, tea, and cola. Parents/guardians can reasonably arrange some useful daily activities according to themselves or the school's online courses, and establish a conducive communication atmosphere so as to prevent unnecessary exaggerations of the illness. Parents/guardians should encourage their children to take enough exercises as well as enough sleep since they can boost body immunity.

## Conclusions

Pediatric neurologists are responsible for the provision of the quality medical services to the epileptic children amid of the outbreak. We encourage them to take advantage of the advanced technology by adopting the online outpatient consultations model and should constantly update themselves with the latest information regarding the management of this illness. Pediatricians should think a better way to handle epileptic children in future outbreaks as they are inevitable.

## Limitations

It worth noting that, these recommendations are made for COVID-19 and cannot be used beyond the outbreak. These recommendations might be applicable in China settings only as other counties don't use the distinction of primary, secondary and tertiary medical services. Besides, other countries don't have online drug supply. There is no specific methodology/evidence that was used to develop these recommendations, however, a group of pediatric chief neurologists from Xiangya Hospital, Central South University participated in making them according to their expert opinions and current literatures.

## Data Availability Statement

The original contributions presented in the study are included in the article, further inquiries can be directed to the corresponding author.

## Author Contributions

BC and MK reviewed the literature, drafted, and wrote the manuscript. SC and JX assisted in reviewing the literature. LW, XD, LY, and FH shared their expert opinions and revised the manuscript for intellectual content. FY and JP conceptualized the study, led the literature search, provided their expert opinions, and critically reviewed manuscript for intellectual content. All authors contributed to the article and approved the submitted version.

## Conflict of Interest

The authors declare that the research was conducted in the absence of any commercial or financial relationships that could be construed as a potential conflict of interest.
